# High Performance of Commercial PAC on the Simultaneous Desulfurization and Denitrification of Wastewater From a Coal-Fired Heating Plant

**DOI:** 10.3389/fchem.2022.916368

**Published:** 2022-07-22

**Authors:** Jiancong Liu, Ying Wang, Yangyang Wang, Zhigang Wang, Xiaoshu Wang, Fanrong Kong, Zheng Yan, Tong Li, Lei Wang

**Affiliations:** ^1^ School of Environment and Energy, Peking University Shenzhen Graduate School, Shenzhen, China; ^2^ School of Construction and Environmental Engineering, Shenzhen Polytechnic, Shenzhen, China; ^3^ State Key Joint Laboratory of Environment Simulation and Pollution Control, School of Environment, Tsinghua University, Beijing, China; ^4^ School of Resources and Environmental Engineering, Wuhan University of Technology, Wuhan, China; ^5^ Chongqing Zhongli Environmental Protection Co., Ltd., Chongqing, China; ^6^ Chinese Society for Environmental Sciences, Beijing, China; ^7^ School of Environment, Northeast Normal University, Changchun, China

**Keywords:** wastewater, desulfurization, nitrate removal, poly aluminum chloride, natroalunite

## Abstract

The flue gas desulfurization wastewater is highly saline and has too many refractory pollutants to be recycled during the desulfurization process of the coal-fired heating plant. Given that waste heat is abundant in coal-fired heating plants, a thermal treatment method was developed to simultaneously remove sulfates and nitrates from the wastewater, with the production of chemical-grade natroalunite and recycled water. The results showed that sulfates and nitrates were 50.3 and 10 g/L in the wastewater, respectively, and only 2.8% and 9.1% were removed after direct treatment at 270°C for 7 h; but these rates increased to 99.3% and 99.9%, respectively, with the addition of commercial poly aluminum chloride. Mass balance summarized that the treatment of 1 ton wastewater needed 0.1 ton PAC and produced 0.11 ton natroalunite and 0.92 ton recycle water. The removal of sulfates and nitrates was mainly done by the precipitation reaction of sulfates such as natroalunite and the redox reaction between nitrates and organics, respectively. Thermodynamic analysis demonstrated that the precipitate reaction occurred at 45°C and accelerated in the temperature range of 45–270°C, but became slow with the decrease of sulfate and Al concentrations in wastewater. Four other reagents were also used for wastewater treatment in comparison with PAC and showed the following order of performance: PAC > citrate calcium > limestone > subacetate aluminum > citrate ferric. This method provided a practical route to treat wastewater from flue gas desulfurization without generating secondary waste.

## 1 Introduction

Coal-fired heating plants are widespread in cold regions of cities/towns, with the mass consumption of high-quality coal every year. Consequently, coal-fired flue gas is discharged, alongside the release of NO_x_ and SO_2_ from the burning of N/S-bearing impurities in coal (Cho et al., 2017; Conidi et al., 2018). These levels of NO_x_ and SO_2_ are at high risk of causing serious photochemical pollution (Ma et al., 2012; Cho et al., 2017) and are legally removed using the selective catalytic reduction process (SCR) in wet flue gas desulfurization systems (Mukherjee et al., 2016; Gingerich et al., 2018), accompanying the discharge of flue gas desulfurization wastewater (Solińska and Bajda, 2022). This wastewater is highly saline, and legal regulations require it be treated and completely recycled in an SCR system without any discharge (Conidi et al., 2018; Anderson et al., 2021; Solińska and Bajda, 2022).

In the past decades, the limestone–gypsum method was developed and applied to the desulfurization of wastewater (Mukherjee et al., 2016). With this method, the wastewater was treated and then recycled several times in desulfurization (Conidi et al., 2018), but inevitably two acknowledged drawbacks emerged. One of which was the production of large amounts of yellowish gypsum. The gypsum contained major impurities such as Fe, Al, Si, and P (Lee et al., 2012; Liu et al., 2016), and subsequently, its comprehensive utilization was limited, leading to the stacking of gypsum waste in a hazardous warehouse (Gingerich et al., 2018). The other drawback was the accumulation of nitrate and halite in the wastewater as the wastewater recycling continued (Xin et al., 2020), which made it highly corrosive to SCR equipment and inappropriate for reuse. This wastewater was finally disposed of by two methods, the flue gas evaporation method (Shuang c hen et al., 2016; Cui et al., 2020) and the low-temperature concentration method (Li et al., 2016; Zheng et al., 2019), separately. For the former, the wastewater was directly discharged into flue gas at 140–400°C, where water was completely evaporated, and the remaining salt was captured by flue dust and then collected in a bag filter (Shuang c hen et al., 2016). For the latter, low-temperature energy was used to heat wastewater, and when steam was released, the impure salt was left behind (Li et al., 2016; Zheng et al., 2019). The heating energy was consumed in the evaporation processes, the recycled water, and the valuable product were not generated. Compared with these methods, membrane capacitance deionizing, electrodialysis, and reverse osmosis processes were effective to remove impure salts and thus recycle water (Cui et al., 2017; Conidi et al., 2018; Anderson et al., 2021), with the high energy consumption and costly membranes. Given that waste heat is abundant in coal-fired heating plants, its comprehensive utilization for desulfurization and denitrification of wastewater should be considered.

It is reported that nitrates and sulfates cannot be decomposed at room temperature, and can be reduced to nitrogen gas and elemental sulfur by microbes via the biological denitrification route [xx], for example, in municipal wastewater treatment plants. However, the flue gas wastewater has high salinity and lacks organics, and it cannot be treated *via* the biological method because the high salinity raises the osmotic pressure of microbes, inhibiting their growth and the organics are important electron donors to biologically reduce sulfates and nitrates (Huo et al., 2020). Thus, the thermal treatment of flue gas wastewater was noteworthy. Compared with sulfates, nitrates are strong oxidants and can be applied in wet oxidation for the treatment of refractory wastewater (Zhu et al., 2019). This wet oxidation is especially accelerated in the presence of metal cations. For instance, by introducing glucose and ferric ions, the redox reaction between nitrates and glucose was initiated and rapidly equilibrated in 3–7 h (Lin et al., 2019), while the remaining nitrate was less than 1 g/L. Nitrate removal can be achieved effectively, but the synergic removal of sulfates has been rarely reported until now. Except for the precipitation of sulfates as low-valued gypsum, the value-added sulfate-bearing deposit was also not reported.

Herein, real flue gas desulfurization wastewater was treated *via* a thermal precipitation route with the addition of commercial poly aluminum chloride (PAC). PAC is a common flocculant in the coagulation treatment of wastewater but shows attractive potential for wastewater purification at high temperatures. With this method, high concentrations of sulfates and nitrates were synergically removed, with the generation of chemical-grade natroalunite. The thermodynamic mechanism of sulfate removal was analyzed, and PAC’s performance was also investigated in comparison with those of four common desulfurization reagents.

## 2 Materials and Methods

### 2.1 Wastewater Composition

The wastewater was categorized as concentrated wastewater in the flue gas desulfurization station of Jinli Gold and Lead Group Co., LTD. (Henan, China). It was at pH 0.4 and contained 50.3 g/L sulfates, 10 g/L nitrates, 16.4 g/L chlorides, and 20.6 g/L sodium, with the low concentrations of 171.6 mg/L TOC, 79 mg/L Fe, and 171 mg/L Ca. Heavy metals and aluminum were not detected in the wastewater. The wastewater was recycled several times *via* the conventional limestone–gypsum method and became highly saline. Thereafter, it was discharged into the flue gas system and rapidly vaporized *via* the flue gas evaporation method, in which inorganic salt was recrystallized, ground to dust, and then co-captured using a bag filter.

### 2.2 Wastewater Treatment

**SCHEME 1:**
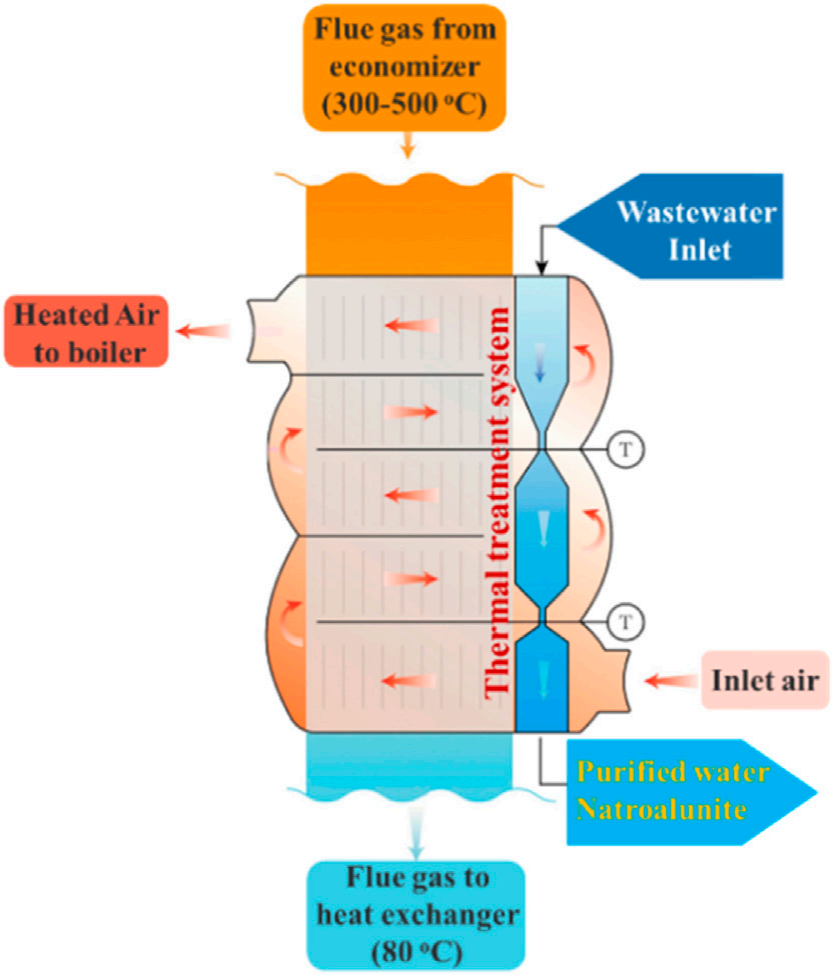
Illustration graph of the thermal module for wastewater treatment.

The flue gas desulfurization station was an ancillary facility of the coal-fired heating plant of Jinli Co., Ltd., and was situated behind the flue gas cooling and dedusting system. When the heating system started, fresh air was blasted into the air pre-heater, in which its temperature was increased from outdoor temperature to nearly 350 C before entering the hearth of the coal-fired heating system. Given that high-temperature energy was recycled by the economizer, the remaining thermal energy of medium and low temperatures was abundant and surplus in the following air pre-heater. Thus, a thermal treatment system was developed for the wastewater treatment and inset the air pre-heater (Scheme 1). The system contained three parts. The wastewater was pumped into the top part, where sulfates and nitrates were removed. It flowed into the middle part and bottom part in turn, where the wastewater was cooled down to below 80°C and further to room temperature. After that, a whitish deposit was generated and collected, and the remaining supernatant was recycled for the next round of flue gas desulfurization.

To optimize the removal efficiencies of sulfuric and nitrate from the wastewater, the corresponding reagent and operation parameters were investigated at a lab scale as follows. A total of 20 ml wastewater was mixed with 0.5 g limestone to form a mixture, and then heated in a Teflon vessel at 270°C for 20 h. After that, the vessel was cooled down to below 30°C and opened, where the deposit and supernatant were collected separately. A control experiment was also performed according to the aforementioned steps, in which limestone was replaced by commercial polymeric aluminum (PAC, Element Guixi, and China), and the corresponding PAC dosage, temperature, and time-course were also optimized. In addition, to avoid the addition of exogenous chloride, organic-metal compounds, for example, subacetate aluminum, citrate ferric, and citrate calcium, were also considered to replace limestone in the removal of sulfates and nitrates from wastewater.

### 2.3 Characterization

The wastewater and corresponding supernatants were characterized by ICP-OES, I.C., pH meter, and TOC analyzer. The generated deposits were also analyzed by SEM and XRD. HSC chemistry software was used to calculate the Gibbs free energy of deposit formation in the thermal system.

In addition, the removal efficiencies of sulfate and nitrate are calculated according to the following equation.

$$R=\left(C_{0}/C_{t}\right)/C_{0}\times 100\%$$ (1)

where *R* is the removal efficiency (%), *C*_*0*_ and *C*_*t*_ are the initial concentrations of sulfates and nitrates, and the concentrations after reaction for t time in the raw flue gas wastewater (mg/L), respectively.

## 3 Results and Discussion

### 3.1 Removal Efficiencies

**FIGURE 1:**
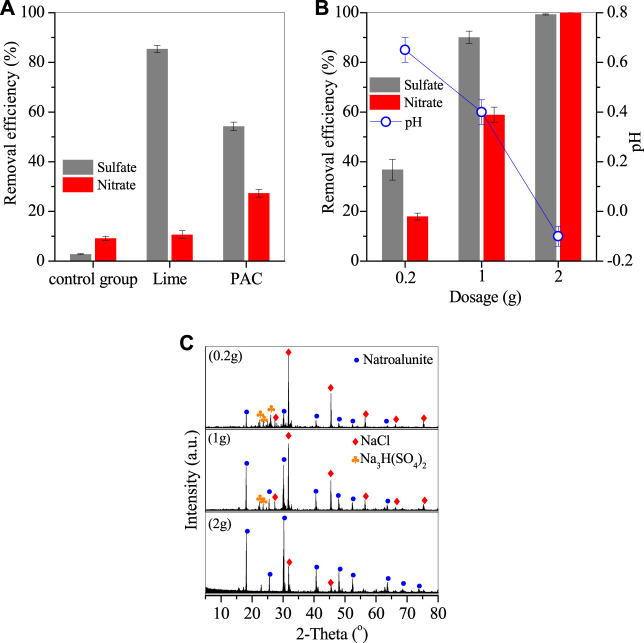
Removal efficiencies of nitrates and sulfates. **(A)** Comparison of lime and PAC with the same dosage of 0.5g, **(B)** optimization of PAC dosage and the corresponding pH variation, and **(C)** XRD patterns of deposit generated by adding PAC (conditions: treated at 270°C for 20 h).

A lab-scale experiment was performed to investigate the removal efficiencies of sulfates and nitrates in the thermal system, as shown in Figure 1. Only 2.9% sulfates and 9.1% nitrates were removed after thermal treatment without adding any reagent because of the thermal decomposition of sulfates and nitrates (Figure 1A). When 0.5 g lime was added, the removal efficiency of sulfates apparently increased to 85.4% due to the formation of gypsum (Liu et al., 2016; Gingerich et al., 2018), but that of nitrate was only slightly elevated to 10.6%. However, by adding 0.5 g PAC, the removal efficiencies of sulfates and nitrates were 54.2% and 27.3%, demonstrating that PAC promoted the synergy removal of sulfates and nitrates. This removal mechanism is detailed in the Section 3.3.

**FIGURE 2:**
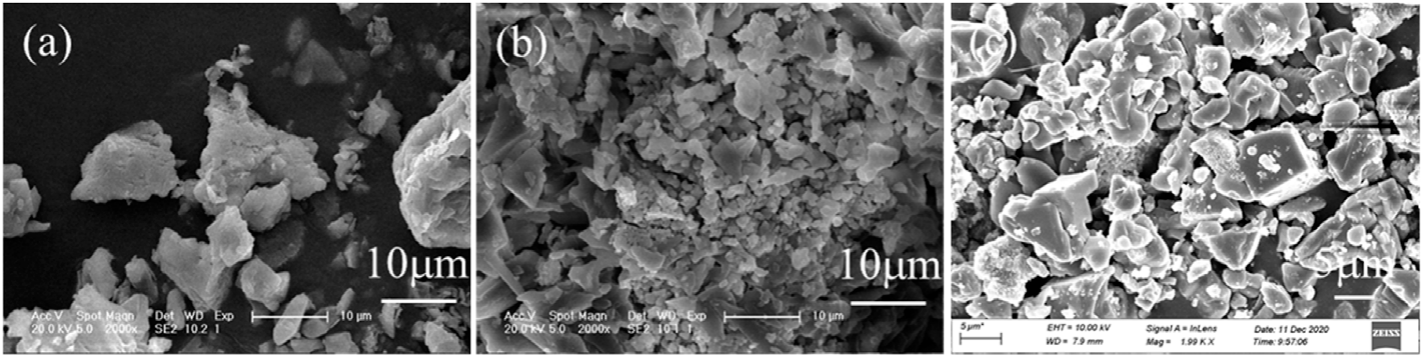
SEM images of deposit generated by adding **(A)** 0.2, **(B)** 1, and **(C)** 2 g PAC.

PAC dosage was also optimized, as shown in Figure 1B. The removal efficiencies of sulfates and nitrates were 36.8% and 17.9% for 0.2 g PAC and steadily increased to 90.1% and 58.9% for 1 g PAC, and further to 99.3% and nearly 100% for 2 g PAC. This demonstrated that PAC had high efficiency in the removal of sulfates and nitrates. The raw wastewater was at pH 0.4, but its pH value was apparently increased to 0.6 with 0.2 g PAC with the removal of sulfates and nitrates, and then steadily dropped to 0.4 and −0.1 by adding 1 and 2 g PAC. The corresponding deposits were generated and showed typical peaks of natroalunite, alongside the peaks of NaCl and sodium hydrogen sulfate (Figure 1C). It is clear that the peak of natroalunite became sharp with the increase of PAC dosage. Accordingly, the deposits were irregular particles for 0.2 g PAC (Figure 2A) and 1 g PAC (Figure 2B), but a portion of them was in the form of broken regular hexahedral particles with a side length of 8 μm by adding 2 g PAC (Figure 2C). During thermal treatment of wastewater, sulfates was involved in the formation of natroalunite, but the nitrate-bearing product was not found in the generated product, suggesting that the nitrate removal was probably assigned to the chemical decomposition, not the precipitation reaction.

**FIGURE 3:**
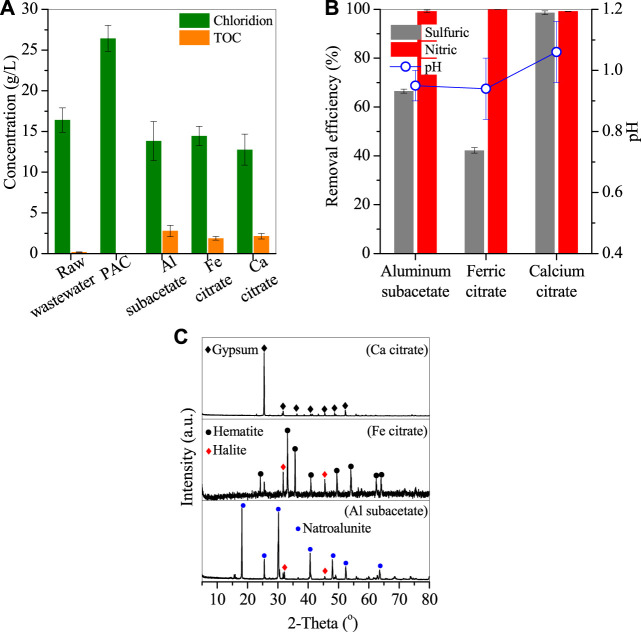
**(A)** The concentrations of chlorion and TOC, **(B)** removal efficiencies of sulfates and nitrates, and **(C)** XRD patterns of deposits generated by adding organic Ca/Fe/Al (conditions: treated at 270°C for 7 h by adding 2 g reagents).

2 g PAC showed optimal performance in the removal of sulfates and nitrates, and after treatment, TOC was below the detectable limit, but the chloride concentration apparently increased from 16.4 to 26.5 g/L due to the addition of commercial PAC (Figure 3A). To avoid the introduction of chloride, subacetate aluminum was used in comparison with citrate ferric and citrate calcium. By adding 2 g subacetate aluminum, sulfate removal only achieved 66.5%, while the removal efficiency of nitrate reached nearly 100% (Figure 3B), with the formation of well-crystallized natroalunite particles (Figure 3C, Al subacetate). While adding 2 g citrate ferric, the removal efficiency of nitrates also reached nearly 100%, but that of sulfates dropped to 42.2% (Figure 3B). However, the corresponding precipitate showed that only the peaks of hematite and halite appeared, and no sulfate/nitrate-bearing compounds were observed. In terms of the 42.2% sulfate removal, these sulfates were probably involved in the natrojarosite precipitation (McCollom et al., 2014). The natrojarosite was weakly crystallized, and its peaks were overlapped by hematite and halite. When 2 g citrate calcium was added, the removal efficiency of sulfates increased to 98.6%, apparently higher than that of subacetate aluminum and citrate ferric, and that of nitrate also reached 99.2%. But yellowish gypsum was generated (Figure 3C, Ca citrate) and belonged to a low-value byproduct (Shuang c hen et al., 2016; Fu et al., 2018). It is noted the wastewater pH was close to 1 with subacetate aluminum and citrate ferric but slightly increased to 1.1 with citrate calcium (Figure 3B). Such pH variation was derived from the involvement of H^+^ in the redox reaction between organics and nitrates, and the Al/Fe hydrolysis (detailed in Section 3.3). After treatment, the rest chloridion did not vary apparently in the wastewater, but the TOC considerably increased to 2.8 g/L for subacetate aluminum, 1.9 g/L for citrate ferric, and 2.1 g/L for citrate calcium (Figure 3A), demonstrating that the added organics were kept in the wastewater. These remaining organics had a potential volatilization risk at high temperatures (Furrer et al., 1998) and polluted the denitrification catalyst when the treated wastewater was recycled for flue gas desulfurization. Thereby, commercial PAC was a desirable reagent in the removal of sulfates and nitrates without the retention of any organics.

### 3.2 Optimization of Temperature and Time

**FIGURE 4:**
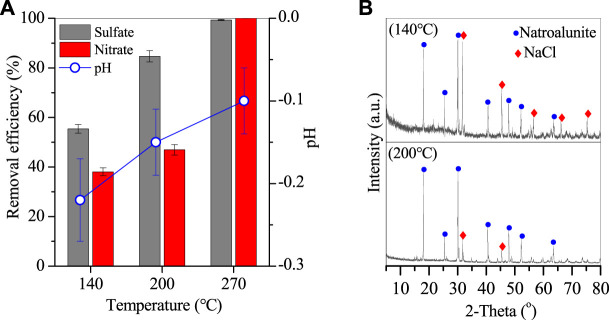
**(A)** Removal efficiency of sulfates and nitrates and corresponding variation of solution pH, and **(B)** XRD of Al-bearing precipitates (conditions: treated for 20 h with 2 g PAC).

**FIGURE 5:**
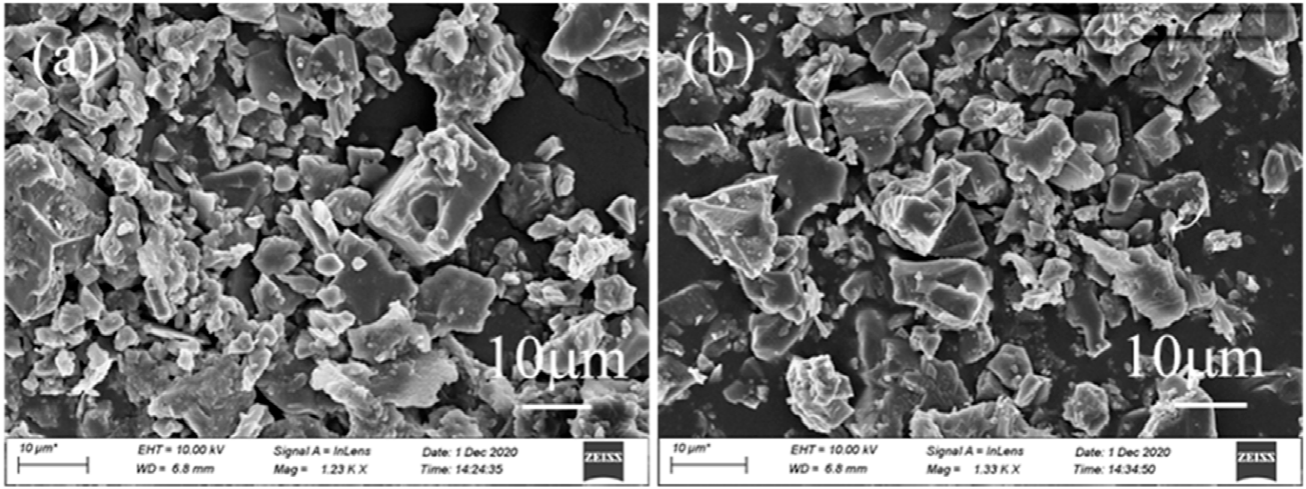
SEM images of deposit generated at **(A)** 140°C and **(B)** 200°C.

Given that PAC was effective in the removal of sulfates and nitrates from wastewater, the corresponding reaction temperature and time were also optimized as shown in Figures 4, 5. With the temperature increasing from 140 to 270°C, the corresponding removal efficiencies of sulfates and nitrates also elevated from 55.4% and 38.1% to 84.7% and 47%, and 99.3% and nearly 100% (Figure 4A), revealing that temperature was an important parameter for sulfate and nitrate removal. With sulfate and nitrate removal, the solution pH slightly increased from −0.2 to −0.1 (Figure 4A), which was generated from the H^+^ consumption in the nitrate removal. After the reaction, an Al-bearing product was also generated and showed typical peaks of natroalunite (Figure 4B) and morphology of broken hexahedral particles, a precursor of that at 270°C (Figure 5).

**FIGURE 6:**
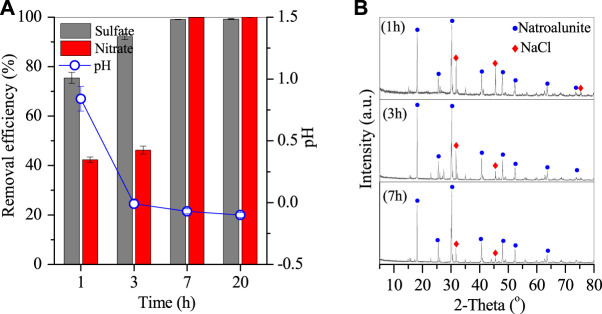
**(A)** Time-course of final pH of the remaining solution and removal efficiencies of sulfates and nitrates, and **(B)** XRD patterns of Al-bearing precipitates (conditions: treated at 270°C with 2 g PAC).

**FIGURE 7:**
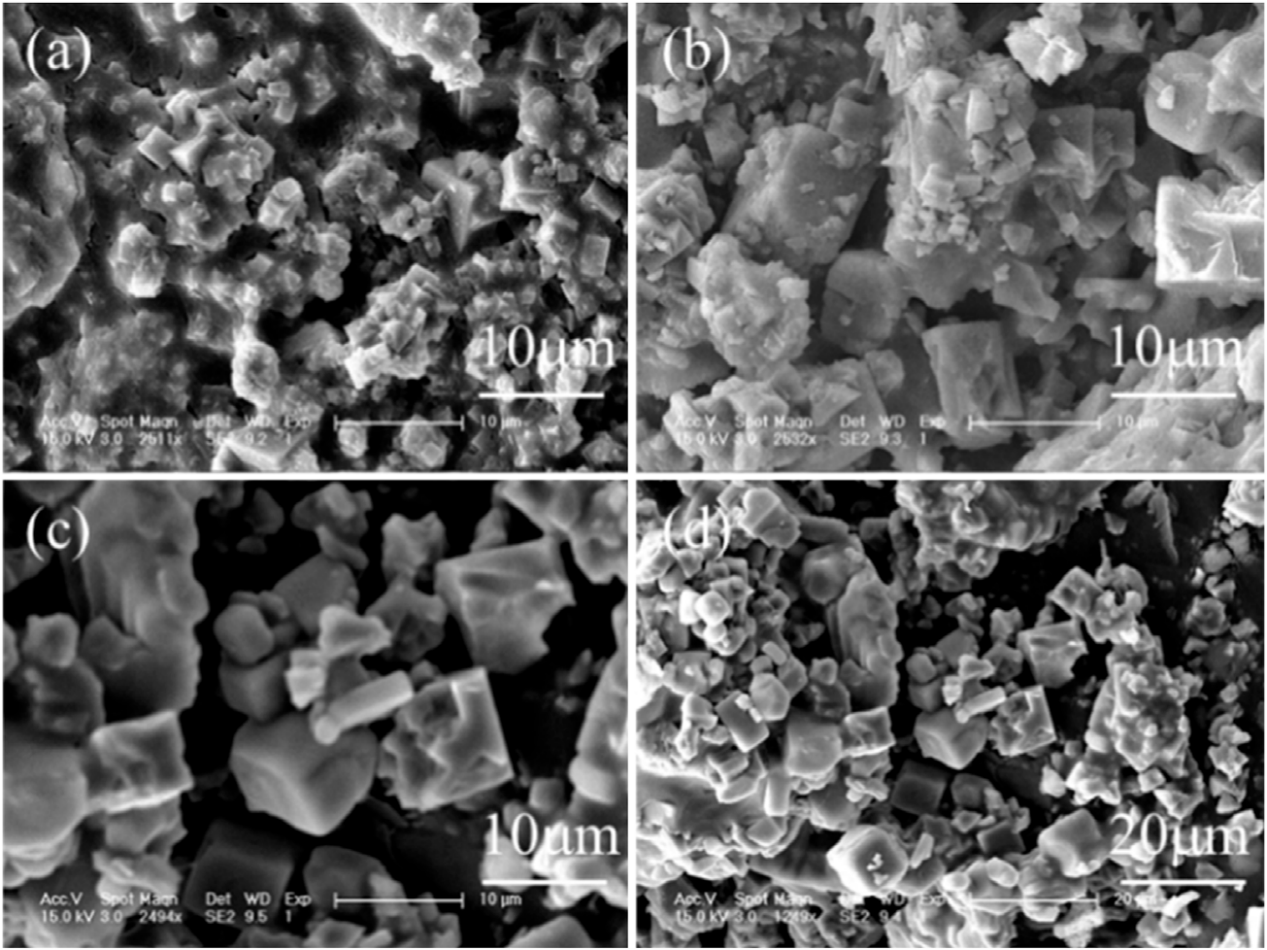
SEM images of Al-bearing deposit generated at **(A)** 1, **(B)** 3, and **(C,D)** 7 h.

The removal of sulfates and nitrates was rapid in the thermal system. Figure 6 (a) shows that the sulfates and nitrates were removed by 75.4% and 42.3% in the initial first hour, and then 92.1% and 46.2% at 3 h, but 99.1% and nearly 100% at 7 h, and stayed almost unchanged at 20 h. The corresponding pH of wastewater increased rapidly to 0.8 in the first 1 h but constantly dropped to −0.1. After reaction for 7 h, the remaining sulfates and nitrates were only 0.45 and 0.009 g/L, and thus 7 h of reaction time was optimal. The generated products were also characterized by XRD and SEM Figures 7A–D. The XRD spectra showed the peaks of natroalunite and halite. The formed product was cubic particles with a side length of 1–3 μm after 1 h (Figure 6A), and grew gradually with the time-course increasing from 1 to 7 and 20 h (Figures 6B,C, 2C).

### 3.3 Removal Mechanism

The wastewater had high salinity and contained 50.3 g/L sulfates, 10 g/L nitrates, 16.4 g/L chloridion, and 20.6 g/L sodium, along with the minimum of TOC, Fe, and Ca. When it was directly treated in the thermal system, a portion of nitrates was also decomposed as NO_x_/N_2_ and H_2_O (Wu et al., 2012; Zhu et al., 2020), with the consumption of H^+^ *via* Eq. 2. Given that the wastewater contained 171.6 mg/L TOC, the redox reaction between nitrates and organics also took place (Eq. 3) (Bian et al., 2020; Su et al., 2020), which accelerated the nitrate removal. By adding 0.5 g limestone, the precipitation reaction of sulfates to Ca^2+^ was spontaneous and accelerated at high temperatures (Fu et al., 2018), leading to increased sulfate removal. Accordingly, nitrate removal was also elevated due to the introduced organics with the addition of limestone. However, without the addition of limestone, extra organics were not introduced, and accordingly, the nitrate removal did not increase apparently. Compared with limestone, the commercial PAC was a mixture comprising 21.6 Al%, 12.8% Cl, 3.3% C, and 23% water content. When 0.5 g PAC was dispersed in the wastewater, fresh organics were introduced and then involved in the redox reaction of nitrate, resulting in the improvement of nitrate removal efficiency. Even though Ca was not found in PAC, Al was abundant and then reacted with sulfates in the thermal system to precipitate as crystallized natroalunite (Eq. 4). However, 10.1 g/L chlorides was introduced with the addition of PAC and was not involved in the precipitation reaction, with the accumulation in the treated wastewater. The amount of added chloridion was apparently less than that of removed sulfates and nitrates. Thus, PAC did have a positive effect on the removal of sulfates and nitrates in the thermal system, where approximately 100% sulfates and nitrates were removed with the addition of 2 g PAC.

$$2NO_{3}^{-}+4H^{+}\to 2NO_{2}^{-}+2H_{2}O$$ (2)

$$3.6H^{+}+3.6NO_{3}^{-}+C_{6}H_{8}O_{7}\to 1.8N_{2}+6CO_{2}+5.8H_{2}O$$ (3)

$$Na^{+}+3Al^{3+}+2SO_{4}^{2-}+6H_{2}O\to NaAl_{3}{\left(SO_{4}\right)}_{2}OH_{6}+6H^{+}$$ (4)

$$NaAl_{3}{\left(SO_{4}\right)}_{2}OH_{6}\to 3AlOOH+Na^{+}+2SO_{4}^{2-}+3H^{+}$$ (5)

**FIGURE 8:**
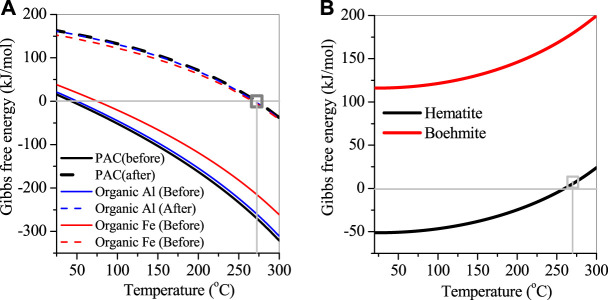
Fitted plot of Gibbs free energy versus temperature. **(A)** before and after the reaction, **(B)** hematite and boehmite.

The thermal performance of natroalunite formation was also analyzed, as shown in Figure 8A. When the reaction temperature was increased up to 45°C, the precipitation of sulfates and Al as natroalunite occurred, along with the consumption of Na^+^ and the release of H^+^ to wastewater. This precipitation reaction accelerated with the temperature increasing from 45°C to 270°C and attained equilibrium at 270°C with the decrease in sulfate and Al concentrations. This was affiliated with the variation of the corresponding Gibbs value from −269.3 kJ/mol before reaction to −0.07 kJ/mol after reaction at 270°C (Figure 8A). After the reaction, the wastewater was cooled down to room temperature. After the reaction, natroalunite was stable and did not convert into highly crystallized boehmite (Eq. 5), even though the Gibbs value of boehmite formation was decreased from 175.3 to 116.3 kJ/mol. Thereby, mass production of natroalunite was collected.

Organic metals, for example, subacetate aluminum, citrate calcium, and citrate ferric, also showed high removal efficiencies of sulfate and nitrate. As the organics were introduced, the reduction of nitrates by organics continued, resulting in high removal efficiency of nitrates, even if organics were overdosed and contributed to the increase of TOC in the treated wastewater. The Al, Ca, and Fe in the organic metals also played different roles in the sulfate removal. The former two metals were involved in the formation of natroalunite (Figure 8A) and gypsum as described previously, but the latter was related to the natrojarosite formation (Eq. 6) and further converted into hematite. The conversion of natroalunite to boehmite showed a positive Gibbs value of >116.3 kJ/mol in the temperature range of 20°C–270°C and was stable in the thermal system. However, natrojarosite showed a chemical structure similar to natroalunite and was stable at 270°C. But it was converted to hematite at the temperature <260°C and accelerated with the temperature decreasing from 260°C to 20°C, in accordance with the variation of Gibbs value from 6.1 kJ/mol at 270°C to −51 kJ/mol at 20°C (Figure 8B). It is noted that the conversion of natrojarosite to hematite only occurred on the precipitate surface, where sulfates were released into wastewater, and then the adjacent Fe atoms replenished hydroxy groups. Thereby, the conjunction of adjacent Fe atoms as Fe–O–Fe bond via hydroxy bond happened, with the release of H^+^ and water molecules *via* Eq. 7. As the Fe–O–Fe bond formation continued, the free channel of sulfates from inside to the surface shrinks, leading to the retardation of sulfate release. Such performance also decreased the removal efficiency of sulfates by organic Fe. In summary, the removal of sulfates and nitrates can be apparently improved by the continuous increase of organic Al/Fe dosage, but the remaining organics were accumulated in the wastewater and contributed to the increase of TOC, which was unacceptable for recycling wastewater.

$$Na^{+}+3Fe^{3+}+2SO_{4}^{2-}+6H_{2}O\to NaFe_{3}{\left(SO_{4}\right)}_{2}OH_{6}+6H^{+}$$ (6)

$$NaFe_{3}{\left(SO_{4}\right)}_{2}OH_{6}\to 1.5Fe_{2}O_{3}+Na^{+}+2SO_{4}^{2-}+1.5H_{2}O+3H^{+}$$ (7)

The wastewater pH also varied in the thermal system, which is mainly derived by two routes. One of which belonged to the redox reaction between nitrates and organics. For instance, to remove 3.6 mol nitrate, 1 mol citric acid and 3.6 mol H^+^ were consumed according to the Eq. 2. This led to an increase in wastewater pH. The main mechanism for nitrate removal was affiliated to the redox reaction between nitrate and organics in the flue gas wastewater, with the consumption of H^+^ (He et al., 2017; He et al., 2021). This raised the pH value of wastewater and also accelerated the precipitation of sulfates as natroalunite. This redox reaction was relatively slow at low temperatures and became intense as the temperature increased to 270°C. The other route was assigned to the precipitation of Al/Fe as natroalunite and natrojarosite with the release of abundant H^+^. Accordingly, the wastewater pH steadily dropped as the precipitation reaction continued. The two reactions equilibrated the wastewater pH. By adding organic metals, the added organics were overdosed, and thus more H^+^ was consumed, leading to the increase in wastewater pH.

### 3.4 Potential Application

**FIGURE 9:**
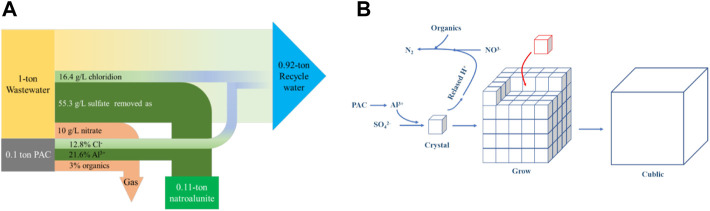
**(A)** Mass balance of sulfate and nitrate removal in the wastewater; **(B)** schematic diagram of sulfate and nitrate removal from flue gas wastewater.

Given that abundant waste heat was not collected in the coal-fired heating system and discharged as vapor via a chimney, such waste heat can be recycled to heat wastewater from a flue gas desulfurization station. This endowed a new thermal route to treat the wastewater, where sulfates and nitrates were synergically removed by adding commercial PAC, and the remaining water could be recycled in the next round of gas desulfurization. To treat 1 ton wastewater needed 0.1 ton commercial PAC, with the production of 0.11 ton highly crystallized natroalunite and 0.92 ton recycle water. Moreover, 0.05 ton sulfates and 0.01 ton nitrates were removed from wastewater (Figure 9A), with the new introduction of 0.009 ton chloride. Furthermore, the mechanism of sulfate and nitrate removal from flue gas wastewater was clarified (Figure 9B). The future investigation should focus on the synthesis of new PAC comprising nitrates and organics without any chlorides. With the use of such a reagent, chloride was not added to wastewater, and the introduced nitrate was completely consumed by organics in the thermal system, without the residue of nitrates and organics in treated water. In parallel, the treated water was adjusted to the neutral condition.

The product natroalunite comprised 91.5 wt% NaAl_3_(SO_4_)_2_OH_6_ and the major impurities were 1.6% Cl and 0.5% Ca, with 5.2% water content, which meet the grade II of chemical APIs and are widely used for the production of sodium sulfate, alums, and molecular sieve (Drouet et al., 2004; Wang et al., 2010; Paulino et al., 2018). Given that the waste heat was free in the thermal power plant, only the consumption of 0.1 ton PAC reagent for 1 ton wastewater treatment is considered with the generation of 0.11 ton natroalunite product, thereby amounting to a total cost of US$ 15.7. It is also noted that the natroalunite product was marketable. When the wastewater was treated using the conventional method with the addition of limestone, nearly 0.08 ton commercial limestone was consumed in the treatment of 1 ton wastewater, with the production of 0.26 ton dihydrate gypsum. However, the product dihydrate gypsum was of low value, and commonly treated by landfills according to the regulation of the local government, so nearly US$ 17.2 should be added. The total cost of using limestone is approximately US$ 27.9/ton. This indicated the economic merit of using PAC in wastewater treatment. Thus, it was a high-value chemical product and showed economic value compared with the conventional product of gypsum from the limestone process.

## 4 Conclusion

A thermal system was designed to treat real wastewater generated from the flue gas desulfurization station in a coal-fired heating plant. Such wastewater contained 50.3 g/L sulfates, 10 g/L nitrates, and 16.4 g/L chlorides. After treatment with the addition of commercial PAC, nearly 100% nitrate was reduced to nitrogen gas, and 99.8% sulfate was precipitated as natroalunite particles, but a portion of chloride was introduced, leading to the increase in chloridion concentration from 16.4 to 26.5 g/L. Despite the accumulation of chloridion concentration, the salt concentration was notably decreased, and the remaining wastewater can also be used as recycled water in the next round of flue gas desulfurization. The generated natroalunite particles contained 91.5% NaAl_3_(SO_4_)_2_OH_6_, and were a commercial chemical product. Such a system had advantages in producing recycled water and natroalunite products and showed desirable application prospects in flue gas desulfurization stations.

## Data Availability

The original contributions presented in the study are included in the article/Supplementary Material; further inquiries can be directed to the corresponding authors.
